# Epicatechin Reduces Striatal MPP^+^-Induced Damage in Rats through Slight Increases in SOD-Cu,Zn Activity

**DOI:** 10.1155/2015/276039

**Published:** 2015-08-02

**Authors:** M. Rubio-Osornio, E. Gorostieta-Salas, S. Montes, F. Pérez-Severiano, C. Rubio, C. Gómez, C. Ríos, J. Guevara

**Affiliations:** ^1^Laboratorio Experimental de Enfermedades Neurodegenerativas, Instituto Nacional de Neurología y Neurocirugía, Manuel Velasco Suárez, 14269 Mexico City, Mexico; ^2^Departamento de Neuroquímica, Instituto Nacional de Neurología y Neurocirugía, Manuel Velasco Suárez, 14269 Mexico City, Mexico; ^3^Departamento de Farmacología, Facultad de Medicina, Universidad Nacional Autónoma de México, 04510 Mexico City, Mexico; ^4^Departamento de Bioquímica, Facultad de Medicina, Universidad Nacional Autónoma de México, 04510 Mexico City, Mexico

## Abstract

Parkinson's disease is a neurodegenerative disorder characterized by movement alterations caused by reduced dopaminergic neurotransmission in the nigrostriatal pathway, presumably by oxidative stress (OS). MPP^+^ intrastriatal injection leads to the overproduction of free radicals (FR). The increasing formation of FR produces OS, a decline in dopamine (DA) content, and behavioral disorders. Epicatechin (EC) has shown the ability to be FR scavenger, an antioxidant enzyme inductor, a redox state modulator, and transition metal chelator. Acute administration of 100 mg/kg of EC significantly prevented (*P* < 0.05) the circling MPP^+^-induced behavior (10 *μ*g/8 *μ*L). Likewise, EC significantly (*P* < 0.05) reduced the formation of fluorescent lipid products caused by MPP^+^. MPP^+^ injection produced (*P* < 0.05) increased enzymatic activity of the constitutive nitric oxide synthase (cNOS). This effect was blocked with acute EC pretreatment. Cu/Zn-dependent superoxide dismutase (Cu/Zn-SOD) activity was significantly (*P* < 0.05) reduced as a consequence of MPP^+^ damage. EC produced a slight increase (≈20%) in Cu/Zn-SOD activity in the control group. Such effects persisted in animals injured with MPP^+^. The results show that EC is effective against MPP^+^-induced biochemical and behavioral damage, which is possible by an increase in Cu/Zn-SOD activity.

## 1. Introduction

Parkinson's disease (PD) is a neurodegenerative disorder characterized by deficiencies in dopaminergic neurotransmission of the ascending nigrostriatal pathway. PD is the second most common neurodegenerative disorder after Alzheimer's disease, reaching millions around the world, prevalently in people over 60 years of age [1]. The main clinical features in patients with PD are resting tremors, rigidity, bradykinesia, and loss of postural stability [2]. Several theories propose that dopaminergic neuronal death is the result of oxidative stress (OS), mitochondrial dysfunction, and iron deposits (Fe) in the* Substantia nigra pars compacta* (*SNpc*) [1]. Oxidative stress is associated with mitochondrial dysfunction and the concomitant overproduction of radical anion superoxides (^∙^O_2_^−^), along with decreased levels of reduced glutathione (GHS) in the SNpc [3]. Accumulation of Fe in the same region contributes to the overproduction of free radicals (FR), since the reaction between ferrous iron (Fe^2+^) and hydrogen peroxide (H_2_O_2_) leads to the formation of hydroxyl radicals (^∙^OH) via Fenton's reaction [4]. This generates lipid peroxidation and neuron death [5]. Intrastriatal injection of 1-methyl-4-phenylpyridinium (MPP^+^) in rats reproduces the main biochemical characteristics of PD. MPP^+^ is incorporated into dopaminergic terminals via the dopamine reuptake transporter. MPP^+^ neurotoxicity is based on the complex I inhibition of the mitochondrial electron transport chain, leading to FR overproduction, lower oxidative phosphorylation, and ATP levels [6]. The entry of Ca^2+^ through the NMDA receptor channel stimulates NO^∙^ overproduction by nNOS [7]. The NO^∙^ readily reacts with ^∙^O_2_^−^ to generate ONOO-, thus promoting tyrosine residue nitration [8] in proteins such as tyrosine hydroxylase (TH), which results in diminished enzymatic activity of TH, which reduces striatal DA content and generates behavioral alterations [9, 10].

Polyphenols are present in high concentrations in fruits, vegetables, and beverages such as green tea and red wine, contributing to their beneficial effects [11]. Epicatechin (EC) is a polyphenol, a secondary metabolite produced in plants from the biosynthetic pathway of flavonoids [12]. It has been suggested that the flavonoids' neuroprotective effect is due mainly to its ability to trap FR [13] and to chelate transition metals [14]. Moreover, experimental evidence indicates that the flavonoid-metal complex mimics superoxide dismutase [15].

The aim of this study was to determine the effect of EC on both behavioral and oxidative damage induced by MPP^+^. We hypothesize that the administration of EC in rats is able to decrease the effect of free radicals, in response to oxidative stress associated with the microinjection of MPP^+^ in the rat striatum.

## 2. Materials and Methods

### 2.1. Experimental Procedures

#### 2.1.1. Animals, Epicatechin, and MPP^+^ Injection

The use and care of animals were done according to official regulatory guidelines (NOM-62-ZOO-1999). Male Wistar rats (280–300 g) were used throughout the study. The animals were housed in acrylic box cages and placed under constant conditions of temperature, humidity, and lighting (12 h light/dark cycles) and provided with a standard commercial rat chow diet and water* ad libitum*. Rats were administered with a 100 mg/kg oral dose (o.p.) of epicatechin (EC) (Sigma-RBI, St. Louis, MO, USA, Cat. E1753) in 10% DMSO. Five hours after the administration of EC, the animals were infused in the right striatum with 10 *μ*g of MPP^+^ iodide (Sigma-RBI, St. Louis, MO, USA, Cat. D048) in 8 *μ*L of sterile saline (s.s.), under sodium pentobarbital anesthesia (40 mg/kg i.p.). The stereotaxic coordinates were 0.5 mm anterior to bregma, −3.0 mm lateral to bregma, and −4.5 mm ventral to the dura, according to the stereotaxic atlas of Paxinos and Watson [16].

#### 2.1.2. Circling Behavior

Apomorphine-induced circling behavior was assessed in rats as previously described [10]. Six days after MPP^+^ intrastriatal injection, animals were treated with apomorphine (1 mg/kg, subcutaneous) and then placed into individual box cages. Five minutes later, the number of ipsilateral rotations at the lesioned striatum was recorded for 60 min. Rotations were considered as 360° turns and results were expressed as the total number of ipsilateral turns in a one-hour period (turns/h).

#### 2.1.3. Striatal Dopamine Levels Measurement

Animals were sacrificed by decapitation and the striatum dissected twenty-four hours after the circling behavioral test. HPLC with electrochemical detection was used to measure striatal levels of DA. Samples were sonicated in 10 volumes of a perchloric acid-sodium metabisulfite solution (1 M 0.1% w/v) and centrifuged at 10,000 ×g for 10 min, and the supernatant was analyzed. Data were collected and processed by interpolation in a standard curve, as previously described [9]. Results are expressed as *μ*mol of DA per mg of wet tissue.

#### 2.1.4. Fluorescent Lipid Products

The effect of epicatechin on fluorescent striatal lipid product (FLP) formation was evaluated six h after MPP^+^ injection. The striata were homogenized in 2.2 mL of sterile saline. One mL of the homogenate was then added to 4 mL of chloroform-methanol mixture (2 : 1, v/v). Tubes were then capped and vortexed for 10 s and the mixture was ice-cooled for 30 min to allow phase separation. The aqueous phase was discarded and a 1 mL of chloroformic layer was transferred to a quartz cuvette, to which 100 *μ*L of methanol was added. Fluorescence was measured in a Perkin-Elmer LS50B luminescence spectrometer at 370 nm of excitation and 430 nm of emission. The protein content was measured according to the method described by Lowry and colleagues [17]. Results are expressed as arbitrary fluorescence units/*μ*g of protein.

#### 2.1.5. Nitric Oxide Synthase (NOS) Activity

NOS activity was measured based on the stoichiometric conversion of L-arginine to NO and L-citrulline [7], with slight modifications as previously described [18]. A volume of homogenized tissue containing 500 mg of protein was incubated for 30 minutes at 37°C in the presence of 10 mM of L-arginine HCl, 0.2 Ci of [3H]-L-arginine, 1 mM of NADPH, 100 nM of calmodulin, 2.5 mM of CaCl_2_, and 30 mM of tetrahydrobiopterin. To quantify the activity of inducible Ca^2+^-independent NOS, the incubation was performed in the presence of 0.1 mM of EGTA and 0.1 mM of EDTA, without adding CaCl_2_. Reactions were stopped by adding a buffer containing 2 mM of EGTA, 2 mM of EDTA, and 20 mM of HEPES, pH 5.5. The reaction mixture was applied onto a 1 mL column of cationic interchange resin (Dowex-50W), which had been previously equilibrated with the stop buffer. This column retained labeled arginine and allowed [3H]-L-citrulline to elute through the column. [3H]-L-citrulline was quantified using a Beckman LS6500 scintillation counter. Results were expressed as ng [3H]-L-citrulline/500 mg of protein per 30 min.

#### 2.1.6. Superoxide Dismutase (SOD) Activity

The superoxide dismutase activity (SOD) was measured using the xanthine/xanthine oxidase method described in [19]. The striatum was carefully weighed and homogenized (1 : 10 w/v) in a buffer consisting of 20 mM sodium bicarbonate, 0.02% Triton X-100, pH 10.2. After homogenization, 50 *μ*L of clarified supernatant was added to 950 *μ*L of reaction mixture, consisting of 10 *μ*M of sodium azide, 100 *μ*M of xanthine, 10 *μ*M of reduced cytochrome c, and 1 mM of EDTA in 20 mM sodium bicarbonate, 0.02% Triton-X100, pH 10.2. The reaction was initiated by adding xanthine oxidase enzyme and monitored by measuring the changes in absorbance at 560 nm in a Lambda-20 Perkin Elmer UV/Vis spectrophotometer. The samples were carried out in duplicate. Firstly, we analyzed the total SOD activity. Later, the Mn-SOD activity was evaluated by the addition of 5 mM of sodium cyanide to the reaction mixture, to selectively inhibit the Cu/Zn-SOD activity. The difference between total SOD and Mn-SOD reflected Cu/Zn-SOD activity. Results were expressed as the percentage variation compared with the respective control values (international units of SOD activity/g of wet tissue).

#### 2.1.7. Serum Alanine Aminotransferase, Alkaline Phosphatase, and *γ*-Glutamyl Transpeptidase Activity Determination

Serum was obtained by cardiac puncture. Determination of alanine aminotransferase (ALT), alkaline phosphatase (ALP), and gamma glutamyl transpeptidase (*γ*-GT) activity was determined as reported elsewhere [20]. All results are in units of *μ*mol/L/min.

#### 2.1.8. Statistics

Results from the evaluation of circling behavior were analyzed by Kruskal-Wallis' test followed by Mann-Whitney's test. Results from dopamine quantification, lipid peroxidation, nitric oxide synthase activity, superoxide dismutase activity, and hepatic damage markers assays were analyzed by ANOVA followed by Tukey's test.

## 3. Results

### 3.1. Circling Behavior

**Figure 1:**
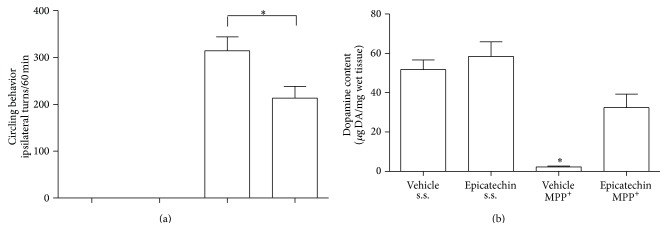
Effect of oral 100 mg/kg epicatechin (EC) on circling behavior and striatal dopamine content in MPP^+^ model. (a) Six days after MPP^+^-induced damage, the EC effect on apomorphine-induced circling behavior was evaluated. The results are expressed as mean ± S.M.E. of 6–8 animals per group. ^*∗*^ *P* < 0.05; data were analyzed by Kruskal-Wallis' test followed by U Mann-Whitney's test. (b) Twenty-four hours after the behavioral test evaluation, dopamine was determined by HPLC coupled to an electrochemical detector. Results are expressed as mean ± S.E.M. of 6–8 animals per group. ^*∗*^ *P* < 0.01, two-way ANOVA followed by Tukey's test.

The evaluation of the apomorphine-induced behavioral test was carried out 6 days after damage induced by MPP^+^. In a pilot experiment to select the dose and the time to inject the EC, we observed that a subchronic oral dose of 40, 60, and 80 mg/kg of EC for 5 days was not able to reduce the turning behavior induced by MPP^+^ (308 ± 28 ipsilateral turns) (data not shown). The subchronic dose of 100 mg/kg for five days reduced the turning behavior after MPP^+^ to 204.1 ± 20.7, ipsilateral turns/60 min. This effect was not different from a similar, previous dose of EC given 5 h before the MPP^+^ injury (data not shown). We therefore evaluated the effect of an acute dose of 100 mg/kg of EC administrated 5 h before the damage was induced by MPP^+^ and then 6 days after the circling behavior was induced by apomorphine. The results indicated that a single acute oral EC dose of 100 mg/kg significantly reduced (*P* < 0.05) the circling behavior (213.3 ± 24.94, ipsilateral turns/60 min) related to the damage induced by MPP^+^ (314.4 ± 29.87, ipsilateral turns/60 min). Single EC oral dose administration partially prevented the unbalanced turning behavior in MPP^+^-treated rats (Figure 1(a)) in the same manner as the chronic oral administration.

### 3.2. Striatal DA Content

Twenty-four hours after the turning experiment, the animals were sacrificed. Striatal tissue was dissected and the DA content was measured. The group treated with the vehicle and saline solutions was considered as the control (51.72 ± 4.92 *μ*g DA/mg wet tissue). EC treatment alone plus saline did not show differences from the control group (58.41 ± 7.4 *μ*g DA/mg wet tissue). In the animals group treated with MPP^+^, a significant (*P* < 0.01) decrease in dopamine content (2.26 ± 0.43 *μ*g DA/mg wet tissue) was found. Whereas the experimental group treated with EC 100 mg/kg plus MPP^+^ prevented the dopamine decay (32.42 ± 6.84 *μ*g DA/mg wet tissue), this result was consistent with the behavioral assay (Figure 1(b)).

### 3.3. Lipid Peroxidation

**Figure 2:**
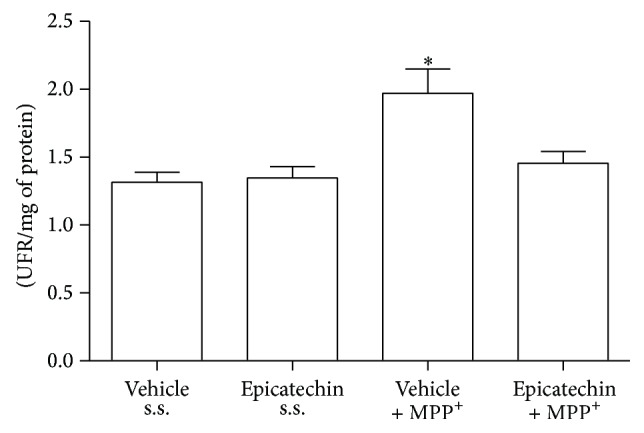
EC reduces MPP^+^-induced striatal lipid peroxidation in rat. Six hours after MPP^+^-induced damage, the formation of lipid fluorescent products was measured as an index of lipid peroxidation. The results are expressed as fluorescence arbitrary units. Each bar represents the mean ± S.E.M. of 8 animals per group. ^*∗*^ *P* < 0.05, two-way ANOVA followed by Tukey's test.

The results obtained from EC administration against MPP^+^-induced oxidative damage are shown in Figure 2. The formation of fluorescent lipid products was measured 6 h after MPP^+^ infusion as a short-term damage marker. Animals treated with the vehicle solution (oral pathway) and sterile saline (intrastriatal) were considered as the control group (1.315 ± 0.073, fluorescent units). We did not find any changes with respect to the control group with EC administration (1.347 ± 0.082) more than with the saline solution. However, MPP^+^ infusion statistically increased (*P* < 0.05) the formation of lipid fluorescent products (1.969 ± 0.179). EC pretreatment prevented the formation of oxidized lipids induced by MPP^+^ to levels shown in the control group (1.455 ± 0.087).

### 3.4. Striatal Nitric Oxide Synthase Activity

**Figure 3:**
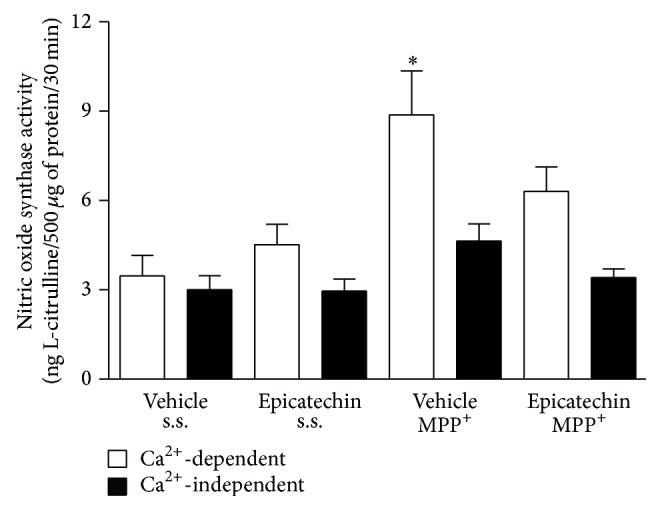
EC effect on striatal Ca^2+^-dependent and Ca^2+^-independent nitric oxide synthase activity. The Ca^2+^-dependent NOS activity was significantly increased after MPP^+^ treatment. The results are expressed as mean ± S.E.M. of 5–7 animals per group in ng L-citrulline/500 mg of protein/30 min. ^*∗*^ *P* < 0.05, two-way ANOVA followed by Tukey's test.

NOS striatal enzymatic activity was also assayed 6 hours after MPP^+^ infusion. The control group showed levels of Ca^2+^-dependent NOS activity (constitutive nitric oxide synthase (cNOS)) (3.46 ± 0.68, ng L-citrulline/500 mg protein/30 min), similar to those of the EC pretreated group (4.51 ± 0.69). However, NOS activity was statistically found to increase (*P* < 0.05) (8.86 ± 1.48) in the MPP^+^-treated group. Acute oral, 100 mg/kg, administration of EC attenuated the effect of MPP^+^ on Ca^2+^-dependent NOS activity (6.30 ± 0.82). Ca^2+^-independent NOS activity remained unchanged in the striatal tissue from all groups studied in this time and no statistical differences were found among groups (Figure 3).

### 3.5. Striatal Superoxide Dismutase Activity

**Figure 4:**
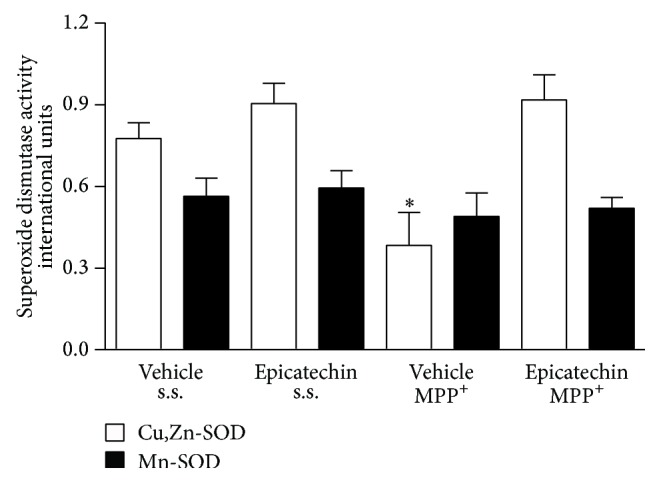
Effect of EC administration on the reduction in Cu/Zn-SOD activity MPP^+^-induced. The infusion of MPP^+^ statistically reduced Cu/Zn-SOD activity (white bars). The Mn-SOD activity did not change among groups (black bars). The results are expressed as mean ± S.E.M. of 6–8 animals per group. ^*∗*^ *P* < 0.05, two-way ANOVA followed by Tukey's test.

Figure 4 shows the results obtained from superoxide dismutase activity within the present paradigm. The white bars represent the results of Cu,Zn-dependent superoxide dismutase (SOD-Cu,Zn) activity. The animals treated with MPP^+^ showed a significant (*P* < 0.05) decrease in superoxide dismutase activity (0.361 ± 0.14), international units, while the rest of the groups remained unchanged (0.774 ± 0.05, vehicle + s.s.; 0.909 ± 0.07, EC + s.s.; and 0.916 ± 0.09, EC + MPP^+^). In this case, it is possible to observe a slight increase of 17.4% in the group treated with EC, more than in the saline solution group, while it is preserved in the experimental group treated with EC + MPP^+^ (18.3%) and Mn-dependent superoxide dismutase (SOD-Mn) activity remained unchanged among groups (black bars).

### 3.6. Liver Damage Indicators

None of the hepatic damage markers analyzed in this study showed a toxic effect after EC administration. The group of animals treated with a single oral dose of 100 mg/kg EC had no effect on ALT, *γ*-GT, and ALP activity when compared with the control group. Likewise, differences were found between the control group treated with vehicle solution and MPP^+^ and the groups treated with EC and MPP^+^ (Table 1).

## 4. Discussion

Parkinson's disease (PD) is a neurodegenerative disorder with increasing incidence as life expectancy increases. There is currently no effective drug therapy to counteract the overproduction of free radicals (FR) and oxidative stress (OS) of the nigrostriatal pathway of patients with this disease. In this study, we initially compared the effect of the oral subchronic administration, of increasing doses (40, 60, 80, and 100 mg/kg), and of epicatechin (EC) with the circling behavior of rats treated with the dopaminergic toxin MPP^+^, in order to determine an adequate EC dose. The results showed that the subchronic administration of 100 mg/kg of EC was able to decrease the ipsilateral turns induced by apomorphine administration in MPP^+^-injured rats. We subsequently evaluated the effect of a single, 100 mg/kg, dose of EC. This dose decreased the circling behavior to the same level that we obtained by the same dose in a subchronic paradigm. This result of the circling behavior was corroborated by quantifying the striatal dopamine content and we found that the effect of a single oral administration of EC was consistent at both levels. The effect of polyphenols extracted from green tea against neurotoxicity induced by MPP^+^ has been related to the blocking of the presynaptic DA transporter (DAT) [6, 21]. However, in this work, the EC exerted partial protection, both behaviorally and in DA content. This evidence suggests that the dose of EC used in this study did not block the DA transporter. Therefore, the effects observed might be the result of some other mechanism.

MPP^+^-induced fluorescent lipid products formation was prevented by EC pretreatment, which presumably counteracted oxidative damage produced by MPP^+^. EC ability to scavenge free radicals appears to be related to the chemical structure of the catechin molecule. A catechol functional group and a hydroxyl group activated a double bond C5 ring neutralizing free radical [22, 23]. Possibly the antioxidant action of EC resembles the action of (−)-epigallocatechin-3-gallate by trapping iron [22], thereby reducing the free iron intraneuronal pool, thus decreasing the overproduction hydroxyl radical (^∙^OH) by the entry of the metal by Fenton's reaction [10]. Given the consistency of our results with the findings reported in the literature, our next goal was to identify the EC action mechanism in the striatum of the rat. We found that EC pretreatment was able to reduce the calcium-dependent nitric oxide synthase activity (cNOS) in MPP^+^-injured rats, indicating that EC has a regulatory effect on the overproduction of ^∙^ON, probably through the ROS-^∙^ON pathway [24]. Interestingly, we observed that EC administration produces a slight increase in Cu,Zn superoxide dismutase (Cu,Zn-SOD) activity, which can be closely linked to its neuroprotective effect. Experimental evidence shows that the formation of flavonoid-metal complexes mimics SOD activity [15]. The overexpression of Cu,Zn-SOD in mice reduces striatal tyrosine hydroxylase nitration and inactivation induced by MPTP administration [25]. This supports the idea of a critical role for superoxide anion in MPP^+^-induced neurotoxicity.

The effect of the acute administration of 100 mg/kg of EC on liver damage was evaluated for the classic markers. No adverse effects were found (Table 1). These results are consistent with the subchronic oral administration of green tea extracts, containing approximately 120 mg/kg of EC, in Wistar rats and showed no adverse effects on the liver [26]. Some reports indicate that the administration of 100 mg/kg of epigallocatechin-3-gallate in mice increases the plasma concentration of alanine transaminase (ALT), while intraperitoneal administration of 150 mg/kg is lethal [27]. Consequently, in the present study we show evidence of the neuroprotective effect of EC on the behavior and oxidative damage of MPP^+^-induced in the rat striatum and on its circling behavior. These changes might be through the induction of an increase of Cu,Zn-SOD activity.

## Figures and Tables

**Table 1:** Serum alanine aminotransferase, *γ*-glutamyl transpeptidase, and alkaline phosphatase as hepatic damage markers. No statistical differences among groups were found.

Treatment	Liver damage indicators		
	ALT	*γ*-GT	ALP
Vehicle + s.s.	20.10 ± 1.90	56.20 ± 4.26	15.54 ± 2.45
EC 100 mg/kg + s.s.	21.28 ± 1.82	66.52 ± 6.21	14.52 ± 1.22
Vehicle + MPP^+^	17.50 ± 1.76	61.27 ± 9.32	13.80 ± 1.09
EC 100 mg/kg + MPP^+^	18.92 ± 2.14	52.53 ± 2.91	12.53 ± 1.20
